# Adaptive Force of hamstring muscles is reduced in patients with knee osteoarthritis compared to asymptomatic controls

**DOI:** 10.1186/s12891-023-07133-y

**Published:** 2024-01-05

**Authors:** Laura V Schaefer, Silas Dech, Friederike Carnarius, Florian Rönnert, Frank N Bittmann, Roland Becker

**Affiliations:** 1 Health Education in Sports, Department of Sports and Health Sciences, University of Potsdam, Potsdam, Germany; 2 Regulative Physiology and Prevention, Department of Sport and Health Sciences, University of Potsdam, Potsdam, Germany; 3 Department of Orthopedics and Traumatology, University Hospital Brandenburg, Brandenburg an der Havel, Berlin, Germany

**Keywords:** Adaptive Force (AF), Maximal isometric Adaptive Force, Holding capacity, Knee osteoarthritis, Maximal voluntary isometric contraction, Strength deficits, Neuromuscular function, Isometric eccentric contraction, Knee flexor muscles, Hamstrings

## Abstract

**Background:**

Quadriceps strength deficits are known for patients with knee osteoarthritis (OA), whereas findings on hamstrings are less clear. The Adaptive Force (AF) as a special neuromuscular function has never been investigated in OA before. The maximal adaptive holding capacity (max. isometric AF; AFiso_max_) has been considered to be especially vulnerable to disruptive stimuli (e.g., nociception). It was hypothesized that affected limbs of OA patients would show clear deficits in AFiso_max_.

**Methods:**

AF parameters and the maximal voluntary isometric contraction (MVIC) of hamstrings were assessed bilaterally comparing 20 patients with knee OA (ART) vs. controls (CON). AF was measured by a pneumatically driven device. Participants were instructed to maintain a static position despite an increasing load of the device. After reaching AFiso_max_, the hamstrings merged into eccentric action whereby the force increased further to the maximum (AF_max_). MVIC was recorded before and after AF trials. Mixed ANOVA was used to identify differences between and within ART and CON (comparing 1st and 2nd measured sides).

**Results:**

AFiso_max_ and the torque development per degree of yielding were significantly lower only for the more affected side of ART vs. CON (*p* ≤ 0.001). The percentage difference of AFiso_max_ amounted to − 40%. For the less affected side it was − 24% (*p* = 0.219). MVIC and AF_max_ were significantly lower for ART vs. CON for both sides (*p* ≤ 0.001). Differences of MVIC between ART vs. CON amounted to − 27% for the more, and − 30% for the less affected side; for AF_max_ it was − 34% and − 32%, respectively.

**Conclusion:**

The results suggest that strength deficits of hamstrings are present in patients with knee OA possibly attributable to nociception, generally lower physical activity/relief of lower extremities or fear-avoidance. However, the more affected side of OA patients seems to show further specific impairments regarding neuromuscular control reflected by the significantly reduced adaptive holding capacity and torque development during adaptive eccentric action. It is assumed that those parameters could reflect possible inhibitory nociceptive effects more sensitive than maximal strengths as MVIC and AF_max_. Their role should be further investigated to get more specific insights into these aspects of neuromuscular control in OA patients. The approach is relevant for diagnostics also in terms of severity and prevention.

## Background

The knee joint is the most affected region of osteoarthritis (OA) with high prevalence and societal relevance [1, 2]. The main symptom is pain [3], but loss of joint functionality, stiffness and swelling are also reported [1, 2, 4]. The etiology and pathogenesis have not yet been clarified, but are considered heterogeneous and very complex [5]. Alongside several identified risk factors like obesity and a history of knee injury [1, 6, 7], stress [8] and muscle weakness [4, 9–11] have also been discussed in the context of OA. According to a recent meta-analysis, knee extensor weakness is associated with knee OA (low quality evidence) [10]. A review on cross-sectional studies reported 10–56% lower isometric and isokinetic concentric knee extensor torque in knee OA patients vs. controls [4]. One study revealed an isokinetic eccentric deficit of 76% in measurements with 90°/s and 180°/s [12].

Increased hamstring muscle activation was found for patients with knee OA compared to controls [13]. According to Hortobágyi et al., this “may interfere with normal load distribution in the knee and facilitate disease progression” [13]. Hamstring muscles are particularly relevant for knee stability due to their synergistic function with the anterior cruciate ligament which impedes anterior tibia shift [14]. However, findings on knee flexor strength deficits range from 4 to 38%, and, thus, are not as clear as for antagonists [4]. Two investigations revealed similar and significant reductions of isometric strength for quadriceps and hamstring muscles (60° or 90° knee flexion) comparing OA patients and controls [15, 16]. Other authors reported non-significant differences in hamstring strength (isometric [17, 18], isokinetic concentric or eccentric [19]).

Due to those inconsistent findings regarding knee flexors, the aim of the present study was to compare various torque parameters of hamstring muscles between male patients with knee OA (ART) and asymptomatic controls (CON). In addition to the assessment of maximal voluntary isometric contraction (MVIC), the Adaptive Force (AF) was also examined. The AF stands for a special neuromuscular function which is considered to be more responsive than pushing actions like the MVIC since it requires more complex neuromuscular control processes [20–22, 22–24]. It assesses the muscular holding capacity by adapting to an increasing external load [20–28]. The length of the muscle tendon unit (MTU) should stay similar thereby while the muscle tension must be immediately and precisely adjusted to the changing external load to maintain the given limb position. From a neurophysiological perspective the sensorimotor control is particularly challenged during such an adaptive holding task. The neuromuscular system has to adapt to the current load and simultaneously has to anticipate the prospective change of the external load. The AF therefore does not only test for muscle strength but also for sensorimotor control. Peripheral sensors (muscle spindle, skin receptors, Golgi tendon organs) detect the current state. In central structures the measured peripheral information is used for actual-target-comparison and the respective efferences are sent to the muscle. The thalamus, cerebellum, inferior olivary nucleus, basal ganglia, cingulate and sensorimotor cortices are especially involved in motor processing [29–48]. Since in those complex control circuitries interfering stimuli are also processed – e.g., emotions in the basal ganglia or cingulate cortex [32, 49], nociception in the thalamus [48] etc. – it is conceivable that the adaptive holding capacity might be especially sensitive to such disruptive inputs. It is hypothesized, therefore, that the maximal holding capacity would be particularly limited in OA patients due to nociceptive afferents [4].

During AF measurement two parameters have to be distinguished: (1) the maximal isometric AF (AFiso_max_; maximal holding capacity) refers to the highest force under holding isometric actions (static conditions) during the external force increase. (2) As soon as the external load exceeds the maximal holding capacity, the MTU starts to lengthen whereby the force increases further until the peak value of one trial is reached (maximal AF (AF_max_); mostly during eccentric action).

AF_max_ was found to be similar to MVIC for elbow extensors in healthy young subjects measured by a pneumatic device, where AFiso_max_ was significantly lower [20]. Therefore, this adaptive holding capacity is considered as a special neuromuscular function which has to be differentiated from other strength capabilities. This is further supported by studies using an objectified manual muscle test (MMT) to assess the AF. A handheld device measures force and limb position during the MMT, whereby the tester applies the external force on the participant’s limb. The participant has the same task as during the pneumatic measurements. Those investigations revealed that AFiso_max_ – in contrast to AF_max_ – was found to be especially sensitive to disruptive stimuli. For example, the holding capacity was significantly reduced in healthy persons perceiving unpleasant odors/imagery vs. pleasant ones or baseline [22–24]. Similar results were found after provoking a slack of muscle spindles (using a specific contraction-shortening procedure or by manipulation) [27, 28]. Moreover, Long COVID patients already showed a reduced holding capacity in input measurements, presumably reflecting their dysfunctional state, which normalized after recovery [50, 51]. A single case showed that nociception might also have a distinct reducing effect on the AFiso_max_, but not on AF_max_. [52]

It is known that joints are richly innervated by neurons (especially the capsule, ligaments, menisci, periosteum, and subchondral bone), the vast majority of which are nociceptors [53]. In OA the innervation of the affected joint might change [53] and an altered peripheral and central sensitivity was found [53, 54]. Even structural changes in areas of the brain were identified in OA (e.g., in the thalamus and cortical gray matter) [54–56]. Considering the structures involved in motor control and processing of nociception as well as the suggested sensitivity of the maximal holding capacity, it is conceivable that the maximal isometric AF might reflect impairments in OA patients more clearly than other common strength parameters. This has been found in our own clinical practice countless times, but appropriate evidence is still pending. Therefore, investigating AF parameters in patients with OA is considered beneficial to gain more information on this specific muscle function and on neuromuscular control in patients with OA.

The main hypothesis of the present study was that the torque parameters MVIC and AF_max_ would be significantly lower in ART vs. CON. This is based on the current knowledge about strength deficits in patients with OA [4, 10, 13, 15, 16]. Since the contralateral knee in patients with OA cannot be considered free of impairments [4], strength deficits were also expected for the less affected/asymptomatic side. Due to the known vulnerability of the holding capacity [22–24, 27, 28, 50–52] a considerably lower AFiso_max_ was hypothesized, especially for the more affected side of ART vs. CON.

Since the AF of hamstring muscles has not been considered before, neither for healthy older persons nor for OA patients, this study should provide first reference values and further insights into OA, especially with regard to diagnostics. Moreover, it should help to clarify the inconsistent findings on knee flexor strength deficits in patients with knee OA.

## Methods

Measurements were performed in one session either at the Neuromechanics Laboratory of the University of Potsdam (Germany) or at the Center for Rehabilitation (Vitalis, Brandenburg, Germany) by the same examiners using the same equipment.

### Participants

A priori sample size analysis was performed with G*Power (V3.1.9.4, Düsseldorf, Germany). Mixed-model analysis of variance (mixed ANOVA; within-between-group interaction) was chosen to compare the torque parameters between ART vs. CON separated by measured side. Due to a previous study investigating AF parameter of elbow extensors [20], large effect sizes were used as a basis. To detect a large effect size of Cohen’s f = 0.4 (α = 0.05; 1 – β = 0.8) a minimum sample size of *n* = 12 per group was necessary. In order to account for possible dropouts (e.g., pain during measurements) 20 patients were included for OA patients and 19 for controls. The inclusion criterion was age between 55 and 80 years for both groups.

Twenty male patients diagnosed with knee osteoarthritis (ART) based on X-ray of grade ≥ 2 (Kellgren-Lawrence Score) on at least one side were recruited from the Department of Orthopedics and Traumatology at the University Hospital Brandenburg a. d. Havel (Germany). Seven patients reported knee complaints on the left, six on the right and seven on both sides. Two patients had total knee arthroplasty (TKA) on one side; hence, this side was excluded from measurements. Eight patients reported knee operations. Four of them were due to OA, the others indicated knee operations before OA onset for other reasons (cruciate ligament, meniscus, tibial plateau).

Nineteen male controls (CON) were recruited from the Brandenburg association for health promotion in Potsdam and from the University of Potsdam (Germany). Inclusion criteria were no knee complaints or any history of knee trauma or surgery.

Demographic data is given in Table 1. Age did not differ significantly between ART vs. CON (*p* = 0.116, unpaired t-test, two-tailed). ART showed significantly higher body mass (*p* = 0.043) and body mass index (BMI) (*p* = 0.003) than CON. Consequently, torque values were normalized to body mass for statistical evaluation.

**Table 1:** Participants' information

	**ART**		**CON**	
n	20		19	
age (years)	66 ± 9		62 ± 6	
body mass (kg)	93.8 ± 19.1^†^		83.0 ± 9.9^†^	
body height (cm)	175.2 ± 6.8		180.7 ± 6.1	
BMI (kg/m^2^)	30.5 ± 5.9^†^		25.6 ± 3.0^†^	
Dominant side (left/right/both)	2 / 13 / 4		1 / 15 / 3	
number of first and second measured sides (left/right)	8 / 10	11 / 9	10 / 8	9 / 8

Anthropometric data, foot preference and number of measured sides for patients with knee osteoarthritis (ART) and asymptomatic controls (CON). Since some limbs had to be excluded (see data processing), the number of measured sides does not always match the sample size n

^†^Significant differences between ART vs. CON: *p* < 0.05

### Clinical examinations and questionnaire

The patients were clinically examined prior to the measurements by an orthopedic surgeon. Furthermore, a trained sports therapist assessed the neuromuscular function of the participants’ hamstring muscles by a manual muscle test (MMT) in the sense of a ‘break-test’ [21–24, 50, 57] before and after the measurement series (positioning on the measurement chair identical to AF measurements). The MMT is used in clinical practice to test the adaptive holding capacity of a muscle. It should provide additional information on the functional state of the measured hamstrings. The tester applies an increasing force in the direction of muscle lengthening where the participant should maintain the starting position isometrically despite the rising external force. The applied maximum force was at a high level but can normally be held by an undisturbed muscle. The MTU is therefore not forced into lengthening in all cases; only if the holding capacity is reduced the muscle merges into eccentric action. If the participant was able to maintain the starting position during the whole force rise the MMT was rated as ‘stable’. If the muscle started to lengthen during the force increase the test was rated as ‘unstable’ [22–24, 50].

Parts of the SF-36 Health Survey [58] were used to inquire about different health aspects especially with regard to knee symptoms.

### Technical equipment and measurement principle

**Fig. 1:**
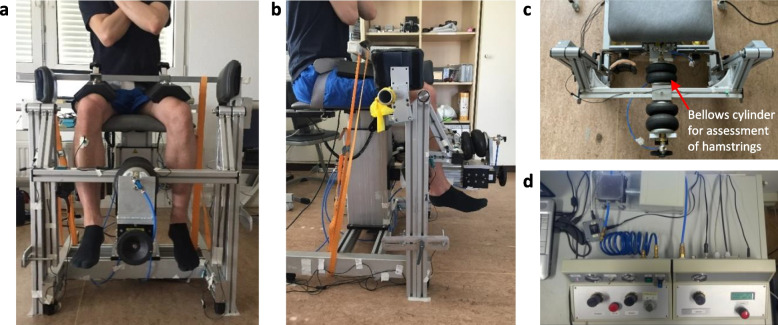
Setting. Pneumatic system for AF measurements of hamstring muscles in (**a**) frontal, (**b**) sagittal and (**c**) transversal view. **d** Control unit and motor-controlled throttle. Note: Air compressor and analog to digital converter are not shown

Figure 1 shows the pneumatically driven system to measure the AF of hamstring muscles. The measurement principle was previously described in detail for elbow extensors [20]. It can detect the AF parameters in a reliable way (ICC = 0.896 – 0.966) with acceptable random errors [20]. Table 2 lists the system components and technical specifications. The system consists of a swing including two levers (left/right; range of motion (ROM): extension-flexion 82°-100°, whereby 0° stands for fully extended knee) which is connected to two bellows cylinders by a cross strut (Fig. 1a, b and c). The bellows cylinders can be pneumatically actuated: one works against the direction of knee extension and the other one against the direction of knee flexion. The latter was used for the present investigation. An interface with a strain gauge on each lever recorded the force between the device and the lower leg. A motor-controlled throttle (Fig. 1d) avoided an abrupt pressure increase at the beginning. Three acceleration sensors (ACC) captured position changes (angles) of each lever (ACC_lever_) and of the participant’s leg (ACC_leg_). The A/D converter buffered data with 1000 Hz recorded by the software NI™ DIAdem 12.0 (National Instruments, Austin, TX, USA).

**Table 2:** Components and technical specifications of the pneumatic AF measuring system

System components (company)	Specification
Compressor (JUN-AIR International A/S, Nørresundby, Denmark)	Model 6, Serial-No. 702997; Condor MDR2 EN 60947–4-1; max. system pressure: 8 bar; calibrated to max. 3 bar
2 bellows cylinders (Zitec Industrietechnik GmbH, Plattling, Germany)	SP-2 B04 R; twofold, Ø 165 mm, max force: 9 kN, stroke length: 1–110 mm (adjustable), rise time: 0.1–30 s continuously
Control unit (Seifert Drucklufttechnik GmbH, Bernsbach, Germany)	Pressure reduction to max. 1 bar
Pressure sensor in control unit (Seifert Drucklufttechnik GmbH, Bernsbach, Germany)	Linear 1 V = 1.05 bar
2 strain gauges and amplifiers (modified by co. Biovision, Wehrheim, Germany)	MLMZ_2000N_67 linear, 1 V = 191.72 N MLMZ_2000N_36 linear, 1 V = 194.72 N
3 acceleration sensors (ACC) (modified by co. Biovision, Wehrheim, Germany)	Sensitivity 312 mV/g (range ± 2g) cosinusoidal, between 70–110° approx. linear, linearity: ± 0.2%
A/D converter (National Instruments, modified by Biovision, Wehrheim, Germany)	14-bit, range: -5 to 5 V
Motor for throttle valve control (RS Components GmbH, Frankfurt/Main, Germany)	Trident DC Geared Motor (12 V dc / 13.9 W, 0.75 Nm, 360 – 1020 rpm)

For AF measurements, the compressed air was directed via the control unit to one bellows cylinder which actuated the swing against the direction of knee flexion. The participant’s task was to prevent the movement of the lever by adapting isometrically to the increasing external load (see below for detailed description). The maximal pressure of the system was adjusted so that each participant was forced into eccentric muscle action thereafter (security stop at 82°). The velocity of airflow into the bellows cylinder was standardized depending on the individual MVIC, so that under stable conditions 70% of the MVIC would be reached after 3 s. The actual velocity depended on the individual ability to adapt to the rising external force. This characterizes the special feature of AF assessment.

### Setting and procedure

After clinical examination, a warmup followed (10 min ergometer bicycling, 1 W/kg; 75 rpm). Subsequently, the participant was positioned in the measurement chair in an upright sitting position with both feet hanging down (hip flexion ~ 90°). Knee joint was flexed in 93° (controlled by hydrogoniometer, 0° refers to fully extended knee joint) and forearms were crossed in front of the chest (starting position of all trials). The rotation center of the participant’s knee (lateral epicondyle) was aligned to the pivot at the hinge of the swing with tensioned hamstrings which should minimize the misalignment of the axes during the contraction phase [59]. To prevent a knee movement out of the pivot during muscle tension, a cushioned fixture was placed on the thighs. The interface was adjusted in height so that it contacted the lower leg most comfortably from posterior between ankle and calf above the Achilles tendon. Lever length was measured from rotational axis to the middle of the interface to calculate torques. One ACC sensor (ACC_leg_) was fixed on the tibial tuberosity with double-sided tape (Fig. 1a).

A specific warmup followed for familiarization with hamstring activation using concentric contractions of the hamstrings in the measurement device (2 × 10 reps, 1 min rest; ROM: from 86° to ~ 92°, end point depended on the force applied by the participant). For this, the pneumatic system was locked in the maximally extended position. The participant pushed the lower leg against the interface in direction of knee flexion (concentric). He was instructed to increase the force up to 50% of the self-estimated maximum. The air in the bellows cylinder was compressed and served as resistance.

Measurements started on the less affected/asymptomatic side of OA patients. For controls the order was randomized. The measurements were guided by two raters (device/software control; instruction/positioning of participant). The following measurement series were conducted for each side:

1. Three MVIC trials: For MVIC-tests the swing was fixed, and the device was passive. The participant was instructed to push against the interface as strong as possible with his posterior lower leg (not explosively; pushing isometric activation of the hamstrings), reach the maximum within 3 s and maintain this for 1 ‒ 2 s. (Resting periods: 60 s).
2. Five AF trials: The pneumatic system was active and provided an increasing load against the direction of knee flexion. The starting position of 93° was adjusted so that the lever was held with 10% of the MVIC. The participant’s task was to maintain this starting position isometrically for as long as possible despite the increasing external load of the system. The pressure continued to rise so that the participant was forced into eccentric action after the maximal holding capacity (breaking point; AFiso_max_) was exceeded. The participant was instructed to decelerate the movement during this lengthening phase, while the force increased further until the maximal AF (AF_max_) was reached. The velocity of MTU lengthening therefore depended on the ability of the participant to decelerate the increasing load. One trial was terminated as soon as the security stop was reached at 82° (resting periods: 120 s).
3. Two MVIC trials: Those were performed again for comparison with the initial MVIC.

### Data processing

Data were processed using NI™ DIAdem 2017 (National Instruments, Austin, TX, USA). Raw signals were filtered: low-pass Butterworth, filter order 10, cut-off frequency 3 Hz (force) or 1 Hz (ACC). Force and ACC signals were converted from volts to Nm and angles, respectively. Torque (Nm) was calculated by τ = r * F, where r refers to the lever and F to force (conversion from V to N see Table 2). Torques were normalized to body mass. The following parameters were extracted for further considerations. Arithmetic means (M), standard deviations (SD), coefficient of variations (CV) and 95%-confidence intervals (CI) were determined for each of the parameter grouped according to ‘less affected/asymptomatic/first measured side of ART’ (ART1), ‘more affected/second measured side of ART’ (ART2), ‘first measured side of CON’ (CON1) and ‘second measured side of CON’ (CON2):

1. MVIC (Nm/kg): the maximum of the three peak values of MVIC tests before and of the two MVIC tests after the AF trials referred to MVICpre and MVICpost, respectively.
2. AF_max_ (Nm/kg): The peak value of each AF trial was determined. The single values of all valid trials were then averaged per participant and side.
3. AFiso_max_ (Nm/kg): the maximal force during holding isometric actions had to be identified for AFiso_max_. For this, no peak (apex) exists in the force curve. The angle signals were used to determine the force at the breaking point from isometric to eccentric muscle action marking the maximal holding capacity (AFiso_max_). Static conditions were defined with a 2°-tolerance. For a detailed description of the algorithm see [20, 60]. Briefly, the latest maximum of angle signals (either ACC_leg_ or ACC_lever_) was determined considering the yielding tolerance (difference of values in direction of knee extension dy < 2°). AFiso_max_ refers to the torque value at the highest curvature (2nd derivation) after the latest maximum of the respective angle signal. The single values of all valid trials were averaged per participant and side.
4. Torque ratios: the ratios $\frac{{AFiso}_{max}}{{AF}_{max}}$, $\frac{{AFiso}_{max}}{{MVIC}_{pre}}$ and $\frac{{AF}_{max}}{{MVIC}_{pre}}$ were calculated to get an impression about the relations of torque parameters.
5. Percentage difference of torques (%Diff, %): For descriptive purposes, the percentage differences between CON and ART (strength deficits) were calculated for each torque parameter by $\%Diff = \frac{M_{ART}-M_{CON}}{M_{CON}}\cdot 100$, where M stands for the arithmentic mean of the respective torque parameter (according to Alnahdi et al. [4]). For within groups, the torque parameters AFiso_max_ and AF_max_ were compared to MVICpre by $\%Diff =\left[\frac{1}{n}\left(\sum^{n}_{i} \frac{{{AFmax}_{i}}{-MVICpre}_{i}}{{MVICpre}_{i}}\right)\right] \cdot100$, i = 1 to n, whereby n stands for the number of participants of each group (ART1, ART2, CON1, CON2; analogues for AFiso_max_).
6. Torque development per degree of yielding (Nm/kg/°): The difference of the angles of ACC_lever_ between start and AF_max_ (ROM, in °) was used to calculate the torque development per degree of yielding ($\frac{{AF}_{max}}{ROM}$).

The following control parameters served the purpose of standardization:

(7) Angle (°): angles of the ACC_lever_ at start and at AF_max_.
(8) Duration of pre-force adjustment: time period (s) of the contraction phase prior to the pressure increase from start of contraction (force signal) to start of pressure rise. Exceptionally long-lasting pre-phases might have influenced the AF.
(9) Durations of isometric and eccentric phases: time periods (s) from start of force increase to AFiso_max_ (isometric) and from AFiso_max_ to AF_max_ (eccentric), respectively.

**Fig. 2:**
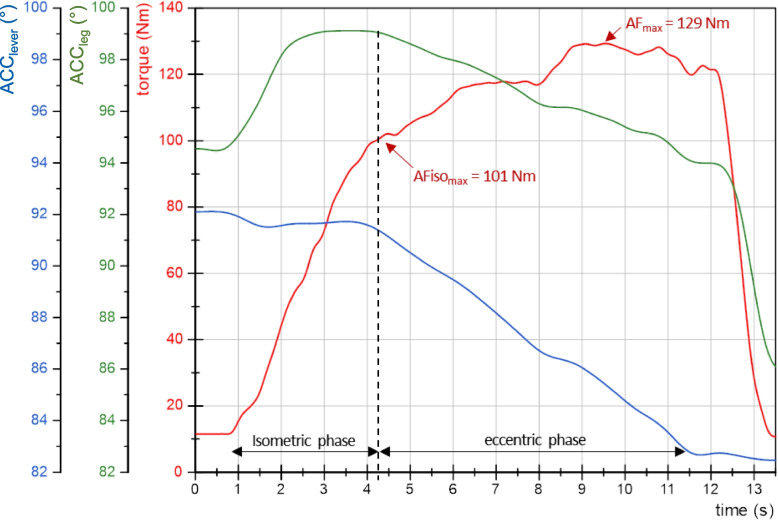
Exemplary curves of torque (red, in Nm) and angles (ACC_leg_ (green) and ACC_lever_ (blue), in °) over time (s) of one trial of Adaptive Force (AF) of the second measured side of a control (m, 56 yrs, 190 cm, 95 kg). The maximal isometric AF (AFiso_max_), the maximal AF (AF_max_) and, accordingly, the isometric and eccentric phases are marked

Figure 2 displays exemplary curves of torque and angles over time of one AF measurement of a control participant. It shows the isometric and eccentric phases as well as the parameters AFiso_max_ and AF_max_. In the beginning the torque increased where the angle of the ACC_lever_ stayed quasi-constant. The initial slight angular rise of the ACC_leg_ (+ 4°) was regularly present, indicating a minor knee flexion. This will be discussed in limitations. Thereafter the angle of ACC_leg_ stayed stable for ~ 2s. The torque value at the highest curvature of the ACC_leg_ at the end of the isometric phase refers to AFiso_max_. Afterwards, the angles of both ACC_lever_ and ACC_leg_ decreased reflecting the eccentric phase. The torque rose further until the peak value of the trial was reached (AF_max_).

### Exclusion of trials for statistical evaluation

For ART, one side of two patients was excluded completely because of TKA. As a consequence, the less affected/asymptomatic/1st-measured side of OA patients (ART1) consisted of 18 limbs (8 left, 10 right) and the more affected/2nd-measured side (ART2) of 20 (11 left, 9 right). For CON, one side of three participants was excluded completely because of existing knee complaints (before measurements) or permanent difficulties in understanding and executing the AF task (e.g., pushing against the lever resulted in invalid trials). In total, 18 limbs (10 left, 8 right) were included for the 1st-measured side of controls (CON1) and 17 (9 left, 8 right) for the 2nd-measured side (CON2). With respect to single trials, all MVIC tests were used for evaluation. Due to unfamiliarity with AF measurements, the first trial of both measured sides was always excluded. In general, any kind of pain during measurements led to exclusion of the respective trial. For that reason, five single trials of four ART patients had to be excluded (pain of knee, hamstrings, lumbar spine). Moreover, single invalid AF trials were excluded, e.g., if participants pushed against the lever (holding task was not executed properly; visible in force signals in 34 of 300 trials) or due to problems during measurements (technical, execution of AF; 12 trials). In total, 67 of 80 AF trials were included for ART2, 64 of 72 for ART1, 59 of 72 for CON1 and 59 of 68 for CON2.

### Statistical analyses

Statistical comparisons were executed in SPSS Statistics 29 (IBM, Armonk, New York, USA). The main focus of the evaluation was on the comparison between OA patients and controls (ART2 vs. CON2 and ART1 vs. CON1). This separation was chosen since the order of measurements might have influenced the results. Moreover, within group comparisons should be considered as well (ART1 vs. ART2 and CON1 vs. CON2). That is why a mixed-model analysis of variance (mixed ANOVA) was executed. As dependent variables the eight main absolute and relative torque parameters were chosen (MVICpre, MVICpost, AF_max_, AFiso_max_, $\frac{{AFiso}_{max}}{{AF}_{max}}$, $\frac{{AFiso}_{max}}{{MVIC}_{pre}}$, $\frac{{AF}_{max}}{{MVIC}_{pre}}$ and $\frac{{AF}_{max}}{ROM}$). One extreme outlier was found for CON2 for $\frac{{AFiso}_{max}}{{MVIC}_{pre}}$. Therefore, the values of this participant regarding AFiso_max_ (AFiso_max_, $\frac{{AFiso}_{max}}{{AF}_{max}}$, $\frac{{AFiso}_{max}}{{MVIC}_{pre}}$) were excluded from further evaluation (that is why CON2 consisted of only 15 participants). Normality of data was assessed with the Shapiro-Wilk-test. All dependent variables were normally distributed, except for $\frac{{AFiso}_{max}}{{AF}_{max}}$ of ART2 (*p* = 0.017). Since ANOVA is considered robust against violations of the normality assumption [61], this ratio was, nevertheless, included. The Mauchly-test of sphericity turned out to be significant, hence, the Greenhouse-Geisser correction was applied (F_G_). Homogeneity of variance (Levene’s test) was fulfilled for each dependent variable except for $\frac{{AF}_{max}}{{MVIC}_{pre}}$ (*p* = 0.010). As this only affected one dependent variable and our main focus was on the post-hoc comparisons to identify differences, we pursued despite this violation [62, 63]. To identify differences between groups a one-way ANOVA was performed [64]. Welch’s ANOVA was chosen since it provides a more robust analysis especially with regard to heteroskedasticity [65]. Tukey’s-HSD was chosen as the post-hoc test due to homogeneity of variance. For within group comparisons (ART1, ART2, CON1, CON2) only the differences between the four absolute torque parameters were included (MVICpre vs. MVICpost, MVICpre vs. AF_max_, MVICpre vs. AFiso_max_ and AF_max_ vs. AFiso_max_). For that a repeated measures ANOVA was executed. Pairwise comparisons were performed with a Bonferroni post hoc correction.

The duration and angles were excluded from mixed ANOVA since they served as preliminary considerations for purposes of standardization; for them, unpaired t-tests (two-tailed) were performed.

For significant pairwise differences, Cohen’s d was calculated from arithmetic means (M) and pooled standard deviation (SD_pooled_) for all significant comparisons using the formula $d= \frac{{M}_{1}-{M}_{2}}{{SD}_{pooled}}$, where ${SD}_{pooled}=\sqrt{\frac{\left({n}_{1}-1\right)\bullet {SD}_{1}^{2}+\left({n}_{2}-1\right)\bullet {SD}_{2}^{2}}{{n}_{1}+{n}_{2}-2}}$ with sample sizes n. Subscripts 1 and 2 correspond to the groups ART and CON or to 1st vs. 2nd measured side, respectively. The effect size Cohen’s d was interpreted as small (0.2), moderate (0.5), large (0.80) or very large (1.3) [66, 67]. The significance level was set at α = 0.05.

## Results

### Outcomes of clinical examinations and SF-36 Health Survey

The onset of knee complaints in ART was 11.5 ± 12.3 years ago (*n* = 14, the other patients made no report). Clinical examination revealed a moderate edema and crepitation for the more affected side (ART2) in two and eight OA patients, respectively. Pain intensity in daily life was 4.32 ± 1.99 on average (VAS; 0 = no to 10 = worst). The activity score of SF-36 with respect to the knee was 63.8 ± 19.8 (0 = activity impossible to 100 = no difficulty in activity). Knee function was 4.9 ± 2.4 (0 = no activity possible to 10 = no restrictions in daily activity). Current health condition was rated 2.42 ± 0.75 (0 = excellent to 4 = bad). General health problems were reported in 18 patients: hypertension (11), obesity (10), heart disease (6), diabetes (4), back pain (5), asthma/lung disease (2), cancer (2), depression (2).

Controls had no knee edema, crepitation or pain. The activity score with respect to the knees was 97.0 ± 5.4, the knee function score amounted to 9.75 ± 0.8 and current health condition was 1.63 ± 0.68. General health problems were reported in 15 controls: hypertension (8), obesity (2), heart disease (1), back pain (6), asthma/lung disease (1).

Regarding the MMT assessment, all hamstrings of CON were rated as stable (CON1: 18, CON2: 15). The hamstrings of ART also showed stability in the majority of MMTs (ART1: 16 stable, 2 unstable; ART2: 15 stable, 3 unstable, 2 unclear).

### Preliminary considerations regarding AF measurements

The duration of pre-force amounted to 10.89 ± 1.64 s, 10.34 ± 1.73 s, 11.33 ± 2.65 s and 10.20 ± 1.84 s for ART1, ART2, CON1 and CON2, respectively, and did not differ significantly between groups (*p* = 0.555 to 0.821). The average duration from start to AF_max_ was 9.0 ± 0.2 s for all groups (CV within groups: 0.19 ± 0.05). The duration of isometric (start to AFiso_max_) and eccentric phases (AFiso_max_ to AF_max_) (Table 3) did not differ significantly between groups (*p* = 0.080 to 0.884). The same applies to the angles at start (corresponds to angle at AFiso_max_) and at AF_max_ (*p* = 0.164 to 0.783) (Table 3).

**Table 3:** Values of assessed parameters

Parameter	ART1	ART2	CON1	CON2
MVICpre (Nm/kg)	0.96 ± 0.33	0.96 ± 0.34	1.38 ± 0.21	1.30 ± 0.30
MVICpost (Nm/kg)	0.90 ± 0.26	0.91 ± 0.31	1.26 ± 0.22	1.25 ± 0.29
AF_max_ (Nm/kg)	0.82 ± 0.31	0.81 ± 0.35	1.21 ± 0.20	1.23 ± 0.28
AFiso_max_ (Nm/kg)	0.60 ± 0.28	0.59 ± 0.34	0.79 ± 0.24	0.98 ± 0.31
AFiso_max_/AF_max_	0.73 ± 0.19	0.74 ± 0.22	0.65 ± 0.15	0.78 ± 0.13
AFiso_max_/MVICpre	0.63 ± 0.21	0.61 ± 0.22	0.57 ± 0.15	0.75 ± 0.16
AF_max_/MVICpre	0.86 ± 0.16	0.84 ± 0.17	0.88 ± 0.09	0.95 ± 0.08
%Diff AF_max_ to MVIC (%)	− 14.35 ± 16.38	− 16.42 ± 17.30	− 12.07 ± 8.80	− 5.06 ± 7.61
%Diff AFiso_max_ to MVIC (%)	− 36.82 ± 21.09	− 38.58 ± 22.07	− 42.68 ± 14.66	− 25.10 ± 15.94
normalized AF_max_ to ROM (Nm/kg/°)	0.12 ± 0.06	0.11 ± 0.05	0.16 ± 0.03	0.19 ± 0.05
angle at start (°)	91.9 ± 0.3	92.0 ± 0.8	92.0 ± 0.3	91.9 ± 0.4
angle at AF_max_ (°)	84.5 ± 1.3	84.3 ± 1.4	83.9 ± 1.1	84.7 ± 1.6
duration isometric phase (s)	4.5 ± 1.7	4.7 ± 2.14	3.8 ± 1.55	5.2 ± 1.5
duration eccentric phase (s)	4.2 ± 2.3	4.1 ± 2.3	5.4 ± 1.5	4.2 ± 2.5

Arithmetic means ± standard deviations of normalized torques (Nm/kg), their ratios, the percentage differences (%Diff) of AF_max_ and AFiso_max_ to MVIC, the torque development per degree of yielding (ratio AF_max_ to ROM; Nm/kg/°), the angles of the ACC_lever_ at start and at AF_max_ as well as the durations of the isometric and eccentric phases of patients with knee OA for the less affected/asymptomatic side (ART1, 1st-measured; *n* = 18) and for the more affected side (ART2, 2nd-measured; *n* = 20) as well as of controls for the first (CON1, *n* = 18) and second measured side (CON2, *n* = 15)

AFiso_max_ = max. isometric Adaptive Force (AFiso_max_); AF_max_ = max. AF; MVICpre = max. voluntary contraction before AF trials; MVICpost = MVIC after AF trials. ROM = range of motion refers to the difference of angles (ACC_lever_) between start and AF_max_

### Torque parameters compared between OA patients and controls

From a descriptive point of view, it should be mentioned that most of the torque parameters showed lower values for ART vs. CON (Table 3). However, CON1 revealed considerably low values for the ratios $\frac{{AFiso}_{max}}{{AF}_{max}}$ and $\frac{{AFiso}_{max}}{{MVIC}_{pre}}$. This is further reflected by the highest %Diff between AFiso_max_ and MVIC for CON1 (− 42.68%), whereas CON2 showed the lowest (− 25.10%); the corresponding values of OA patients were in between (ART1: − 37%; ART2: − 39%) (Table 3).

**Fig. 3:**
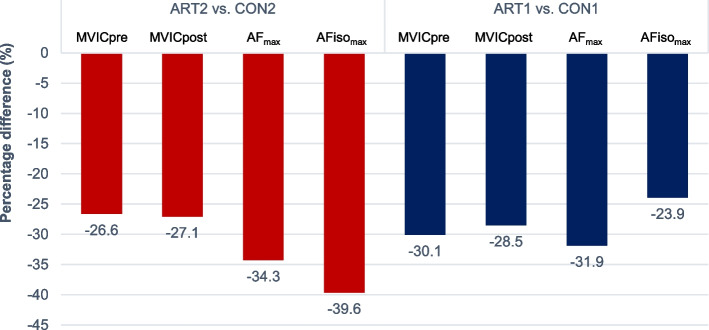
Percentage differences between the more affected side of OA patients (ART2) vs. 2nd-measured side of controls (CON2) (red) and between the less affected/asymptomatic side of OA patients (ART1) vs. 1st-measured side of controls (CON1) (blue) regarding the averaged torque parameters: MVIC before AF trials (MVICpre), MVIC after AF trials (MVICpost), maximal Adaptive Force (AF_max_) and maximal isometric AF (AFiso_max_) (all in %)

The %Diff between ART vs. CON regarding the absolute torque parameters are illustrated in Fig. 3. Noticeable is that AFiso_max_ showed the greatest %Diff for ART2 vs. CON2, whereas it was lowest for ART1 vs. CON1. The other parameters showed rather similar %Diff.

The mixed ANOVA revealed a significant interaction between torques x group (F_G_(6.12, 136.59) = 6.923, *p* < 0.001, partial η^2^ = 0.237). Welch’s ANOVA showed significant differences between the groups (ART1, ART2, CON1, CON2) for MVICpre, MVICpost, AF_max_ and $\frac{{AF}_{max}}{ROM}$ (all *p* < 0.001) as well as for AFiso_max_ (*p* = 0.002), $\frac{{AFiso}_{max}}{{MVIC}_{pre}}$(*p* = 0.023) and $\frac{{AF}_{max}}{{MVIC}_{pre}}$ (*p* = 0.014). Only $\frac{{AFiso}_{max}}{{AF}_{max}}$ missed significance (*p* = 0.075).

**Fig. 4:**
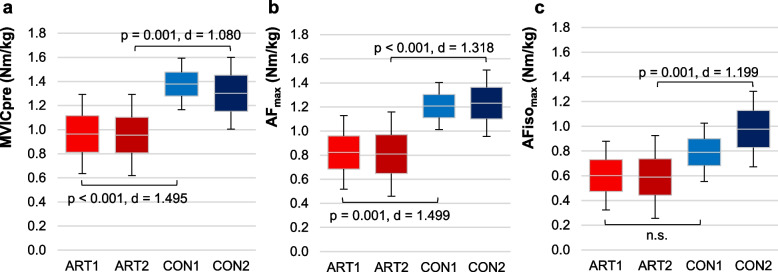
95%-confidence intervals incl. arithmetic means and standard deviations (error bars) of (**a**) the MVIC before AF trials (MVICpre), (**b**) the maximal Adaptive Force (AF_max_) and (**c**) the maximal isometric AF (AFiso_max_) (all in Nm/kg) for ART1 (*n* = 18, light red), ART2 (*n* = 20, dark red), CON1 (*n* = 18, light blue) and CON2 (*n* = 15, dark blue). p-values of Tukey’s-HSD post hoc test and effects sizes Cohen’s d are given in case of significance

**Fig. 5:**
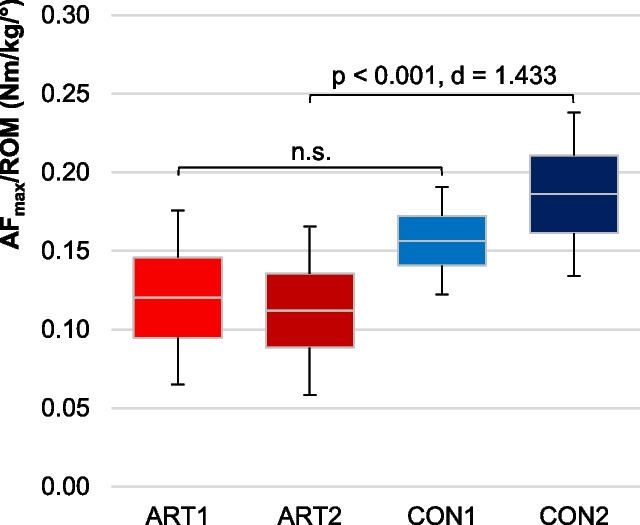
95%-confidence intervals incl. arithmetic means and standard deviations (error bars) of the torque development per degree of yielding (AF_max_/ROM, in Nm/kg/°) for ART1 (*n* = 18, light red), ART2 (*n* = 20, dark red), CON1 (*n* = 18, light blue) and CON2 (*n* = 15, dark blue). p-values of Tukey’s-HSD post hoc test and effects sizes Cohen’s d are given in case of significance

More relevant for the present investigation are the pairwise comparisons between ART2 vs. CON2 and ART1 vs. CON1. The more affected side of OA patients (ART2) showed significantly lower values than CON2 regarding MVICpre, MVICpost, AF_max_, AFiso_max_ and $\frac{{AF}_{max}}{ROM}$ (Table 4, Figs. 4, 5). The torque ratios did not differ significantly between ART2 vs. CON2, however, $\frac{{AF}_{max}}{{MVIC}_{pre}}$ just missed significance (*p* = 0.062) (Table 4).

The less affected side of OA patients (ART1) vs. CON1 showed significantly lower values only regarding MVICpre, MVICpost and AF_max_ (all *p* = 0.001). The other parameters did not differ significantly between ART1 vs. CON1 (Table 4, Fig. 4, 5).

It has to be pointed out that AFiso_max_ as well as the torque development per degree of yielding ($\frac{{AF}_{max}}{ROM}$) differed significantly between patients and controls only for the more affected side, but not for the less affected side of OA patients.

**Table 4:** Pairwise comparisons of torque parameters between and within groups

Dependent variable	Mean difference	Standard error	95% CI lower border	95% CI upper border	Significance p	Cohen’s d
**ART2 vs. CON2**						
MVICpre (Nm/kg)	− 0.38	0.09	− 6.34	− 0.122	**0.001**	− 1.080
MVICpost (Nm/kg)	− 0.37	0.09	− 0.60	− 1.34	**0.001**	− 1.122
AF_max_ (Nm/kg)	− 0.45	0.10	− 0.71	− 0.20	** < 0.001**	− 1.318
AFiso_max_ (Nm/kg)	− 0.39	0.10	− 0.65	− 0.12	**0.001**	− 1.199
AFiso_max_/AF_max_	− 0.05	0.06	− 0.21	0.12	0.879	-
AFiso_max_/MVICpre	− 0.14	0.06	− 0.31	0.04	0.168	-
AF_max_/MVICpre	− 0.11	0.04	− 0.23	0.00	0.062	-
AF_max_/ROM (Nm/kg/°)	− 0.07	0.02	− 0.12	− 0.03	** < 0.001**	− 1.433
**ART1 vs. CON1**						
MVICpre (Nm/kg)	− 0.42	0.10	− 0.68	− 0.15	**0.001**	− 1.495
MVICpost (Nm/kg)	− 0.36	0.09	− 0.60	− 0.12	**0.001**	− 1.504
AF_max_ (Nm/kg)	− 0.39	0.10	− 0.64	− 0.13	**0.001**	− 1.499
AFiso_max_ (Nm/kg)	− 0.19	0.10	− 0.45	0.07	0.219	-
AFiso_max_/AF_max_	0.08	0.06	− 0.07	0.24	0.511	-
AFiso_max_/MVICpre	0.06	0.06	− 0.11	0.23	0.789	-
AF_max_/MVICpre	− 0.02	0.04	− 0.14	0.09	0.957	-
AF_max_/ROM (Nm/kg/°)	− 0.04	0.02	− 0.08	0.01	0.139	-
**ART2 vs. ART1**						
MVICpre (Nm/kg)	− 0.01	0.10	− 0.26	0.25	1.000	-
MVICpost (Nm/kg)	0.01	0.09	− 0.22	0.24	0.999	-
AF_max_ (Nm/kg)	− 0.01	0.09	− 0.26	0.24	0.999	-
AFiso_max_ (Nm/kg)	− 0.11	0.09	− 0.26	0.24	0.999	-
AFiso_max_/AF_max_	0.01	0.06	− 0.15	0.16	0.999	-
AFiso_max_/MVICpre	− 0.02	0.06	− 0.18	0.14	0.992	-
AF_max_/MVICpre	− 0.02	0.04	− 0.14	0.09	0.964	-
AF_max_/ROM (Nm/kg/°)	− 0.01	0.02	− 0.05	0.03	0.954	-
**CON2 vs. CON1**						
MVICpre (Nm/kg)	− 0.05	0.10	− 0.31	0.22	0.969	-
MVICpost (Nm/kg)	0.02	0.09	− 0.22	0.26	0.997	-
AF_max_ (Nm/kg)	0.05	0.10	− 0.20	− 0.31	0.945	-
AFiso_max_ (Nm/kg)	0.19	0.10	− 0.08	0.46	0.267	-
AFiso_max_/AF_max_	0.14	0.06	− 0.03	0.30	0.146	-
AFiso_max_/MVICpre	0.18	0.07	0.00	0.35	**0.047**	− 1.154
AF_max_/MVICpre	0.07	0.05	− 0.05	0.19	0.428	-
AF_max_/ROM (Nm/kg/°)	0.03	0.02	− 0.01	0.07	0.300	-

Tukey’s HSD post-hoc tests between the groups ART2 vs. CON2, ART1 vs. CON1, ART2 vs. ART1 and CON2 vs. CON1 comparing the normalized torque parameters MVICpre, MVICpost, AF_max_ and AFiso_max_ (Nm/kg), their ratios as well as the torque development per degree of yielding (AF_max_/ROM; Nm/kg/°). Given are the mean differences between ART and CON (ART minus CON) as well as between 2nd and 1st measured side for within group comparisons (2nd minus 1st side), their standard errors, lower and upper borders of 95% confidence intervals (CI) as well as the significances p and effect sizes Cohen’s d in case of significance

Parameters: AFiso_max_ = max. isometric Adaptive Force; AF_max_ = max. AF; MVICpre = max. voluntary contraction before AF trials; MVICpost = MVIC after AF trials. ROM = range of motion (angle difference from start to AF_max_). Groups: ART1 = less affected/ asymptomatic side of patients (1st-measured, *n* = 18), ART2 = more affected side of patients (2nd-measured, *n* = 20), CON1 = 1st-measured side of controls (*n* = 18), CON2 = 2nd-measured side of controls (*n* = 15). Significant values are displayed in bold

### Torque parameters compared between measured sides within OA patients and controls

The less vs. more affected side of patients (ART 1 vs. ART2) did not differ significantly in post-hoc tests for any of the parameters (Table 4).

For controls, the 2nd-measured side (CON2) showed higher values than the 1st-measured side (CON1) for all parameters except for MVICpre. Nevertheless, only $\frac{{AFiso}_{max}}{{MVIC}_{pre}}$ was significantly higher for CON2 vs. CON1 (Table 4). The %Diff between AF_max_ and MVICpre was relatively high for CON1 with − 12% whereas it only amounted to − 5% for CON2. Due to this substantial difference an additional paired t-test (two-tailed) was performed which turned out to be significant (t(13) = 2.564, *p* = 0.024, d = 0.685). This indicated that the first and second measured sides of the control group differed with regard to the AF assessment.

### Comparison of torque parameters within each side of OA patients and controls

Pairwise comparisons (Bonferroni corrected) of the absolute torque parameters for each measured side are given in Table 5. It should be emphasized that for all measured limbs MVICpre and AF_max_ were significantly higher than AFiso_max_ with very large effect sizes (d > 1.000). Moreover, only CON2 showed no significant difference between MVICpre and AF_max_. For ART1, ART2 and CON1 significantly higher MVICpre vs. AF_max_ were found, however, with comparatively low effect sizes (d = 0.425 to 0.835). Regarding MVICpre vs. MVICpost, only CON1 showed significantly higher values for MVICpre.

**Table 5:** Pairwise comparisons of torque parameters within each measured side of OA patients and controls

Torque parameters	Mean difference	Standard error	95% CI lower border	95% CI upper border	Significance p	Cohen’s d
**ART1**						
MVICpre vs. MVICpost	0.06	0.03	-0.03	0.15	0.338	-
MVICpre vs. AF_max_	0.14	0.04	0.02	0.26	**0.014**	0.444
MVICpre vs. AFiso_max_	0.36	0.06	0.19	0.53	** < 0.001**	1.190
AF_max_ vs. AFiso_max_	0.22	0.04	0.10	0.35	** < 0.001**	0.758
**ART2**						
MVICpre vs. MVICpost	0.04	0.03	-0.01	0.09	0.191	-
MVICpre vs. AF_max_	0.15	0.03	0.06	0.24	**0.001**	0.425
MVICpre vs. AFiso_max_	0.37	0.05	0.21	0.52	** < 0.001**	1.085
AF_max_ vs. AFiso_max_	0.22	0.05	0.08	0.36	**0.001**	0.639
**CON1**						
MVICpre vs. MVICpost	0.11	0.03	0.03	0.20	**0.008**	0.526
MVICpre vs. AF_max_	0.17	0.03	0.08	0.26	** < 0.001**	0.835
MVICpre vs. AFiso_max_	0.59	0.05	0.43	0.75	** < 0.001**	2.609
AF_max_ vs. AFiso_max_	0.42	0.04	0.29	0.55	** < 0.001**	1.924
**CON2**						
MVICpre vs. MVICpost	0.05	0.03	-0.04	0.14	0.696	-
MVICpre vs. AF_max_	0.07	0.03	-0.01	0.15	0.107	-
MVICpre vs. AFiso_max_	0.32	0.05	0.19	0.46	** < 0.001**	1.075
AF_max_ vs. AFiso_max_	0.25	0.03	0.16	0.35	** < 0.001**	0.872

Bonferroni post-hoc tests of RM ANOVA within the groups ART1, ART2, CON1 and CON2 comparing the normalized torque parameters MVICpre, MVICpost, AF_max_ and AFiso_max_ (Nm/kg). Given are the mean differences and standard errors, the lower and upper borders of 95% confidence intervals (CI), significances p and effect sizes Cohen’s d in case of significance

Parameters: AFiso_max_ = max. isometric Adaptive Force; AF_max_ = max. AF; MVICpre = max. voluntary isometric contraction before AF trials; MVICpost = MVIC after AF trials. Groups: ART1 = less affected/asymptomatic side of patients (1st measured, *n* = 18), ART2 = more affected side of patients (2nd-measured, *n* = 20), CON1 = 1st-measured side of controls (*n* = 18), CON2 = 2nd-measured side of controls (*n* = 15). Significant values are displayed in bold

## Discussion

This study investigated different torque parameters of hamstring muscles in male patients with knee OA compared to asymptomatic controls with special consideration of the AF. Regarding the standardization of AF measurements, it should be noted that the related parameters (angles, durations of pre-force, isometric and eccentric phases) did not differ significantly between groups; hence, the results of the other parameters can be interpreted without concerns.

The most important results were that only the more affected side of OA patients (ART2) showed significantly lower values than controls (CON2) regarding the maximal holding capacity (AFiso_max_) and the torque development per degree of yielding ($\frac{{AF}_{max}}{ROM}$). This was not the case for the less affected/asymptomatic side (ART1) vs. CON1. MVIC and AF_max_ were significantly lower for both sides of OA patients vs. controls.

The maximal holding capacity was assumed to be especially vulnerable to interfering inputs [22–24, 27, 28, 50, 51] and, therefore, was expected to reflect the impairments in OA most clearly. The more affected side of OA patients showed AFiso_max_ deficits of − 40% compared to controls (*p* = 0.001, d = 1.2). For the less affected/asymptomatic side this difference amounted to − 24% (*p* = 0.219). Therefore, the hypothesis of a substantially reduced holding capacity in knee OA can be accepted, especially for the more affected limb. Moreover, AFiso_max_ was the only absolute torque parameter (in Nm/kg) which distinguished the comparisons between the more affected and the less affected side of patients vs. controls. This can be interpreted as an influence of OA on this specific motor function, which might possibly reflect the degree of severity of OA and could, therefore, be used for diagnostics. This is in line with our clinical experience. Not in line with this were the results regarding the torque ratios.

As mentioned in the introduction, previous studies showed reductions of AFiso_max_ related to AF_max_ in reaction to disruptive stimuli from − 39 to − 53%, whereas AF_max_ remained at a similarly high level as for baseline measurements or trials with positive stimuli [22–24, 27, 28, 50, 51]. This was revealed in different settings (objectified manual muscle test in participants with other indications/interventions). However, it reflected a clear reduction of the torque ratios in affected vs. unaffected participants. In the present study, contrary to expectation, those ratios did not reveal significantly lower values for OA patients (affected) compared to controls (unaffected), not even for the more affected side. This might be attributed to the fact that MVIC and AF_max_ were also significantly reduced in OA patients vs. controls (d > 1.000; %Diff ranged from − 27% to − 32%). The more affected side of OA patients (ART2) vs. CON2 showed a trend in accordance with the hypothesis for $\frac{{AFiso}_{max}}{{MVIC}_{pre}}$ with − 18% lower values; however, it missed significance (*p* = 0.168). Specific conditions of the setting, particularly for patients with knee OA, could have influenced these results. This, of course, is speculative; nevertheless, two considerations should be emphasized which have to be discussed controversially. (1) Influence of the measurement position on muscle stability in the sense of AF and (2) Influence of the method of AF assessment (manually vs. device) on strength parameters in affected knees in consideration of the performed repeated maximal contractions. With respect to point (1), from our clinical experience symptomatic OA patients usually show a clear instability during MMT (reflected by a considerably low holding capacity), but maximal strengths are not affected to the same extent. However, in our clinical practice the patients are usually assessed in supine or prone position. In the present study the participants were measured in sitting position, in which almost all hamstrings of patients (31 of 38) showed stable MMTs before measurements. This could be attributable to different initial positions in which the examiner tests the muscle. However, the knee joint angle and lever length were similar, and the examiner positioned himself in a way so that the applied force was appropriate to uncover muscular instability during the MMT. We assume that the sitting position with hanging legs could have generated a traction effect on the knee. This could have reduced inhibiting nociception. As a consequence the holding capacity could have switched from ‘unstable’ to ‘stable’ in the sitting position, preventing the AFiso_max_ from showing even lower values. Such instant changes of the holding capacity are known and can presumably be explained by the complex control processes mentioned in the introduction [22–24, 27, 28, 50, 52]. During standing or walking activities knees are compressed due to gravity. This is assumed to enhance the nociceptive inflow and might, in turn, reduce the muscular holding capacity. Muscular stability in the sense of AF is considered necessary during such activities of everyday life or sports. It is required to provide adequate joint function and to avoid decentering shifts and overload of passive structures. Future investigations in patients with OA should avoid a possible knee strain relief in sitting position to investigate whether stronger deficits of the holding capacity appear under conditions which are closer to everyday life. Moreover, investigations could compare the AF of knee muscles between both conditions, knee strain relief and forced compression.

With respect to point (2), the manual assessment of muscular stability clearly differs from the approach using the pneumatically driven device used in the present investigation. For the latter, maximal intensities are challenged in each trial, especially during the eccentric phase. Moreover, MVIC tests were performed before AF assessment which is not done in our clinical practice. The repeated contractions under maximal intensities might have irritated the structures of the already affected knees even more and/or might have reinforced fear avoidance in OA patients [68]. This might have led to a stronger reduction of torque parameters in patients than in controls, not only regarding the holding capacity but also for the less sensitive MVIC and AF_max_.

Besides these considerations, it was noticeable that controls also showed a considerably low ratio $\frac{{AFiso}_{max}}{{MVIC}_{pre}}$ for the first measured side (CON1) with an average of 57.35%. It amounted to 72% for the second measured side (CON2) and differed significantly between CON1 and CON2. OA patients did not show such a significant difference between the first and second measured sides. Moreover, AF_max_ and MVIC differed significantly for CON1, not for CON2. In OA patients, AF_max_ and MVIC differed significantly for both sides. A previous study using the same device assessing elbow extensors in young healthy participants revealed no significant differences between AF_max_ and MVIC [20]. Hence, AF_max_ and MVIC were hypothesized to show similar torque values. The differences regarding $\frac{{AFiso}_{max}}{{MVIC}_{pre}}$ between the first and second measured side of controls as well as the significant difference between AF_max_ and MVIC for CON1 could speak for familiarization effects regarding the AF assessment. Those did not occur in previous studies [20, 25]; however, they investigated different muscles. The assessment of knee flexors might be more uncommon for participants. Considering the probable familiarization effects, we assume that the second measured side of controls reflects the regular picture of AF parameters in hamstrings of healthy participants. For the same reason, the comparison between the less and the more affected side of OA patients has to be interpreted with caution. Nevertheless, the comparisons between OA patients and controls can be considered reliable, since they were based on similar preconditions with respect to the measured sides.

The second main result was that OA patients could not generate as much force per degree of yielding with their more affected limb compared to controls. $\frac{{AF}_{max}}{ROM}$ showed significantly lower values for ART2 vs. CON2 with a very large effect size (d = 1.433). To the best of our knowledge, no studies using such a parameter exist. The assessment of the so-called rate of torque development during (pushing) isometric muscle actions can be considered similar. This was found to be lower for quadriceps muscle in OA knee patients in one study [69], whereas another study did not find such differences [70]. Although those studies assessed the knee extensors in a different procedure (pushing isometric vs. adaptive eccentric actions), the present results might support the findings of Suzuki et al. [69] by showing lower torque development per degree of yielding for the more affected side of OA patients. A lower force development per degree of yielding means, in turn, a larger giving way in the joint per unit of force increase. Consequently, such behavior must lead to greater instability of a joint under load. This might reflect another aspect of impaired neuromuscular function in OA patients. Motor control deficits are known for patients with knee OA resulting from different nociceptive and non-nociceptive sensory processing [70, 71]. Impaired proprioception has also been reported for OA [72, 73]. This could be a relevant factor for the results found in the present study as well. The AF was suggested to rely especially on a well-functioning proprioceptive system [28]. Since it comprises aspects of strength and proprioception, the investigation of the AF in OA patients might be more beneficial than assessing strength or proprioception separately. A notable feature is that it is closer to real life motions due to the factor of adaptation and likely represents a unique and yet unseen motor output.

Another related aspect which might reflect a probable deficit of neuromuscular function in OA should be pointed out, although the comparison between ART2 and CON2 missed significance (*p* = 0.062). Controls were able to reach 95% of the MVIC during the deceleration of an increasing load (eccentric muscle action; AF_max_), whereas OA patients could only reach ~ 84% of the MVIC. This is in line with Hortobágyi et al. who found highest strength deficits during eccentric actions of quadriceps muscles in patients with knee OA compared to controls [12]. This is presumed to reflect a deficit in reaching the actual available maximal forces during MTU lengthening. Together with the significantly reduced holding capacity and torque development per degree of yielding in the more affected side of OA patients, this might indicate that the ability to appropriately hold and decelerate external loads during adaptive muscle actions is reduced in patients with knee OA. In case the joint angle is increasing but the force development falls behind, the stabilization of the joint could be impaired, i.e., keeping it centered at its pivot. This might result in a higher strain of the joint under load. It is assumed that this increases the risk of complaints, structural degeneration and injury especially during activities including adaptive components (e.g., stair descent, landing). It is known, for example, that non-contact injuries occur during loaded muscle lengthening [74, 75]. However, it is still unclear why these injuries only occur in some individuals or certain situations, where commonly performed movements take place. The holding capacity – with its sensitivity to disruptive stimuli – and the torque development per degree of yielding might help to close the gap of understanding the underlying mechanisms and could support to identify persons at risk. That is why the ability to hold or decelerate varying external loads is considered relevant even before OA develops. The findings indicate that in OA patients not only the load but particularly the load capacity should be considered. The assessment of the adaptive holding capacity could also support and improve exercise therapy. Training under unstable conditions might harm the joint structures due to the assumed misalignment during activities that include holding or decelerating external loads. Therefore, the aim should be to perform exercise therapy under stable conditions. Since the holding capacity can switch immediately from instability to stability if a stimulus is applied that is appropriate to the respective individual, it could help to identify a suitable exercise where the muscles are stable (e.g., under traction of the joint).

Incorporating the results of torque parameters into the current state of research, the MVIC of the measured controls in the presented study (1.36 ± 0.26 Nm/kg; mean of CON1 and CON2) are in line with previous ones reported by Danneskiold-Samsøe et al. [76] They found an average of 1.39 ± 0.19 Nm/kg for isometric knee flexion in age-matched participants (*n* = 30, 64.5 ± 10.1 yrs., range 50 – 79; BMI: 26.4 ± 3.03 kg/m2) [76]. Hence, MVIC values of the older controls in the present study can be regarded as regular. The found strength deficits of knee flexors regarding MVIC in OA patients are in line with some previous investigations [4, 15, 16] and in contrast to studies which revealed no significant differences [17, 19]. As stated by Alnahdi et al. the contralateral knee in patients with OA cannot be considered free of impairments [4]. In the present study, 7 of 20 patients reported bilateral symptoms. Besides the above-mentioned potential familiarization effects, this aspect might explain the non-significant MVIC differences between the more and less affected/asymptomatic side in OA patients. Another possible reason might be a reduced overall strength in both legs of the patients: if one knee is affected the patients might be less active which will also affect the contralateral leg. Considering the long symptomatic periods in ART (averagely 11.5 yrs.), reduced physical activity is conceivable, resulting in lower fitness and increased BMI. BMI differed significantly between ART and CON which supports this hypothesis and previous findings that obesity and OA are correlated. [1]

The general strength deficits which were found for MVIC and AF_max_ for both sides of OA patients might be a common picture. Besides this, it must be highlighted again that the maximal holding capacity as well as the torque development per degree of yielding were significantly reduced only on the more affected side of OA patients vs. controls. Further research could verify if those parameters could serve as special biomechanical markers in diagnostics, reflecting impairments of the neuromuscular control in patients with knee OA, possibly even in terms of severity. This would be especially relevant for the early stages or even the prevention of OA.

### Limitations

Besides the above-mentioned limitations (knee strain relief in sitting position), some participants have reported difficulties in perceiving the increasing load at the contact point of the interface. Thus, the adaptation to the increasing external force might have been delayed resulting in an early deviation of the starting position. Even if a stable position could have been adjusted thereafter at a different knee joint angle, the considered initial value of AFiso_max_ would have been low, nevertheless. The main question, however, was if the participants would be able to maintain the initial static position. Furthermore, the trials in which the participants reported clear execution/adjustment difficulties were excluded from evaluation (invalid trials, see methods); hence, a possible influence of those adjustment difficulties on the results is considered minor.

The angle of the ACC_leg_ showed an initial deviation in the direction of knee flexion in all trials suggesting a shortening of the MTU. The angle of ACC_lever_ stayed stable. This inevitable “misalignment” between the two axes is known and is attributed to “knee-joint kinematics, the compliance of the dynamometer components (seat and attachment pad), and the deformation of the soft tissues.” [59] In the present study the angle of the ACC_lever_ should secure a similar starting position for all participants and the angle of ACC_leg_ was included additionally to determine AFiso_max_. The alignment error should be similar for all participants since the same procedure was used. Since no direct conclusion on the muscle-tendon mechanical properties were relevant (e.g., tendon/fascicle length [59]) and only deviations from the starting angle (relative parameter) were used for analyses, indicating macro changes of the entire MTU, we consider the misalignment between angles of ACC_lever_ and ACC_leg_ as unproblematic for the objective of the study.

A limitation of the algorithm which was used for AF evaluation might be that it does not differentiate between fast and slow yielding. This point was addressed by considering the torque development per degree of yielding. This, however, referred to the lengthening phase and not to the isometric period, where slow and fast yielding was also visible in the ACC_leg_ within the 2° tolerance. Assessing this in the future could provide further relevant information. The algorithm has now been analyzed in more than 800 trials and captures the AFiso_max_ appropriately [20]; hence, possible distorting effects from evaluation are considered minor. Last but not least, the findings could indicate familiarization effects as discussed above. Those were not present in previous studies and, therefore, were not considered necessary before conducting the study. In light of the rather uncommon task, further studies should include a familiarization session.

## Conclusion

The study provided first values of AF for knee flexor muscles in male patients with OA and asymptomatic male controls between 55 and 80 years. Significant strength deficits regarding MVIC and AF_max_ were revealed for both sides of OA patients compared to controls supporting some previous investigations.

The most important findings were the significantly reduced holding capacity (AFiso_max_) and torque development per degree of yielding which were only found for the more affected side of OA patients vs. controls. Those parameters might reflect special aspects of neuromuscular function which could potentially also mirror the degree of severity of OA.

The expected substantial breakdown of the holding capacity in relation to the other strengths (AF_max_, MVIC) did not occur, which is in contradiction to our clinical experience. To ensure this result was not influenced by the measurement setting (sitting position with potential knee strain relief), AF assessment in patients with OA should be executed in different positions.

The findings on the impaired holding capacity, reduced torque development and tendentially reduced exploitation of the individual strength potential (MVIC) during adaptive eccentric actions in OA should be verified in future studies. If the results can be replicated, these parameters could be promising enrichments in the functional diagnosis of OA, also in terms of prevention.

## Data Availability

The datasets used and/or analyzed during the current study are available from the corresponding author on reasonable request.

## Abbreviations

ACC: Acceleration sensor
AF: Adaptive Force
AFiso_max_: Max. isometric AF (holding capacity)
AF_max_: Maximal AF
ART: Patients with knee osteoarthritis
ART1: Less affected/asymptomatic side of OA patients, first measured
ART2: More affected side of OA patients, second measured
BMI: Body mass index
CI: Confidence interval
CON: Age-/sex-matched controls
CON1: First measured side of controls
CON2: Second measured side of controls
CV: Coefficient of variation
M: Arithmetic mean
MMT: Manual muscle test
MTU: Muscle tendon unit
MVIC: Maximal voluntary isometric contraction
MVICpre: Maximum of three MVIC tests before AF trials
MVICpost: Maximum of two MVIC tests after AF trials
OA: Osteoarthritis
ROM: Range of motion
SD: Standard deviation
TKA: Total knee arthroplasty
